# *Gymnadenia conopsea* (L.) R. Br.: A Systemic Review of the Ethnobotany, Phytochemistry, and Pharmacology of an Important Asian Folk Medicine

**DOI:** 10.3389/fphar.2017.00024

**Published:** 2017-02-03

**Authors:** Xiaofei Shang, Xiao Guo, Yu Liu, Hu Pan, Xiaolou Miao, Jiyu Zhang

**Affiliations:** Key Laboratory of New Animal Drug Project, Gansu Province, Key Laboratory of Veterinary Pharmaceutical Development of Ministry of Agriculture, Lanzhou Institute of Husbandry and Pharmaceutical Sciences of Chinese Academy of Agricultural ScienceLanzhou, China

**Keywords:** *Gymnadenia conopsea*, traditional medicine, glucosides, tonifying activity, anti-viral activity

## Abstract

*Gymnadenia conopsea* (L.) R. Br. (*Orchidaceae*) is a perennial herbaceous orchid plant that grows widely throughout Europe and in temperate and subtropical zones of Asia. In China, its tuber has been used in traditional Chinese medicines, Tibetan medicines, Mongolian medicines and other ethnic medicines, and taken to treat numerous health conditions. The present paper provides a review of the traditional uses, phytochemistry, biological activities, and toxicology to highlight the future prospects of the plant. More than 120 chemical compounds have been isolated, and the primary components are glucosides, dihydrostilbenes, phenanthrenes, aromatic compounds, and other compounds. *G. conopsea* and its active constituents possess broad pharmacological properties, such as the tonifying effect, anti-oxidative activity, anti-viral activity, immunoregulatory, antianaphylaxis, antigastric ulcer, sedative, and hypnotic activities, etc. However, overexploitation combined with the habitat destruction has resulted in the rapid decrease of the resources of this plant, and the sustainable use of *G. conopsea* is necessary to study. Meanwhile, the toxicity of this plant had not been comprehensively studied, and the active constituents and the mechanisms of action of the tuber were still unclear. Further, studies on *G. conopsea* should lead to the development of scientific quality control and new drugs and therapies for various diseases; thus, its use and development require additional investigation.

## Introduction

**Figure 1:**
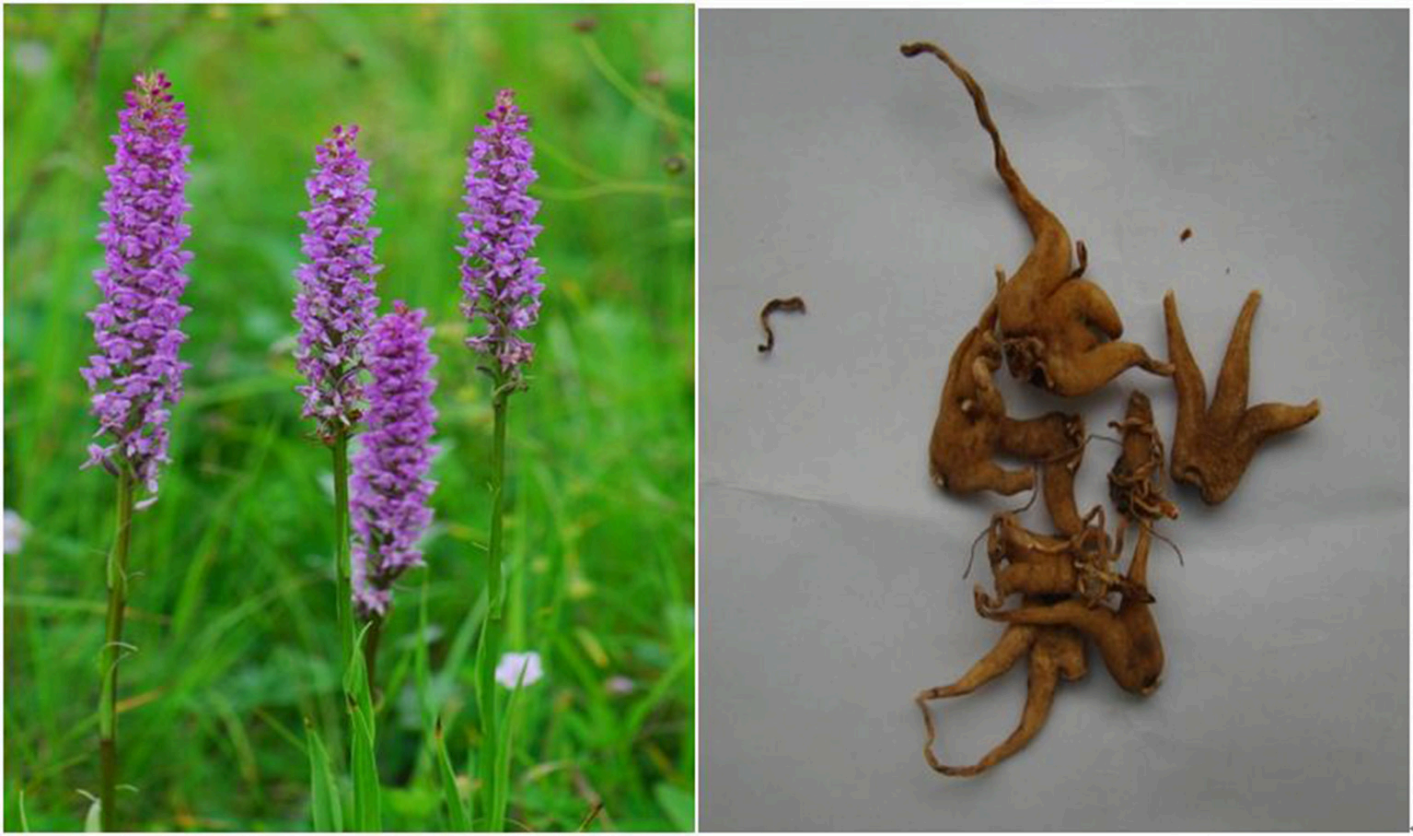
**Photographs of *Gymnadenia conopsea* (L.) R. Br. and the tuber**. We also thanks for the provider of Figure 1, Zhou Yao from http://www.plantphoto.cn.

*Gymnadenia conopsea* (L.) R. Br. (*Orchidaceae*) is a perennial herbaceous flowering plant that is distributed from 200 to 4700 m altitude throughout northern Europe, including England, Ireland, Russia, etc., and temperate and subtropical zones in Asian countries, including Nepal, China, Japan, and the Korean peninsula (Commission of Flora Reipublicae Popularis Sinicae, 2004; http://frps.eflora.cn/frps). For thousands of years, due to the prominent effects on invigorating the spleen, nourishing the lungs and blood, regenerating body fluid, and controlling bleeding with astringents, the tubers of *G. conopsea* was ascribed as a reinforcing agent of traditional medicines in China. It has been primarily used to treat kidney asthenia, cough, and dyspnea induced by lung asthenia, consumption diseases, neurasthenia, chronic diarrhea, morbid leucorrhea, chronic hepatitis, and other diseases in some Asian countries (Chinese Materia Editorial Committee, State Chinese Medicine Administration Bureau, 2002). Because the contour of the tuber is similar to the palm of the human hand, the tuber was given the Chinese name *Shou Zhang Shen*, meaning “ginseng likes palm hands” (Figure 1). In 1977, the tuber of *G. conops*ea was listed in the Pharmacopeia of the People's Republic of China (Committee for the Pharmacopoeia of P.R. China, 1977). Now, it is widely used as a folk medicine and traditional health food by Tibetans, Mongolians, the Han people, and other ethnic groups in China.

Because of the marked therapeutic effects and nutritional actions, researchers have widely investigated the properties of the tuber of *G. conopsea* Modern pharmacological studies have shown that it possesses broad pharmacological properties and can be used in the following treatments: tonifying effect, antioxidative, anti-viral, gastric ulcer prevention, anti-aging, immunoregulatory, antianaphylaxis, sedative, hypnotic, etc. Most of these actions have closely matched traditional uses. The chemical compounds from this plant have also been extensively studied, and glucosides, toluylenes, dihydrostilbenes, phenanthrenes, aromatic compounds, and other compounds have been isolated and identified.

In this review, advances in the ethnobotanical, phytochemical, biological and pharmacological activities, and toxicology of *G. conopsea* are presented and critical assessment. And the data supports its use and exploitation in new drugs.

## Botanical description

Commission of Flora Reipublicae Popularis Sinica According to the description by Meekers et al. (2012) and the Commission of Flora Reipublicae Popularis Sinicae (2004), *Gymnadenia conopsea* (L.) R. Br. is an apolycarpic, perennial, terrestrial, and fragrant orchid herb that belongs to the *Gymnadenia* genus of *Orchidaceae* family, and distributs at forests, grasslands, and waterlogged meadows from 200 to 4700 m altitude. It has about 69 synonyms of this species, but only *Gymnadenia conopsea* (L.) R. Br. is an accepted and approved name in the World. The stem is 20–60 cm, erect, slim, terete, or angled above and leafy with 2–3 brown membranous sheaths at the base. The leaves are green with dimensions of 5.5–15 × 1–2.5 cm, and the lower leaves are erect to slightly spreading, more, or less narrowly oblong-lanceolate or linear-lanceolate, obtuse to subacute, and slightly hooded at the apex, entire, keeled, and folded and have 1–2 or more veins on each side of the midrib; the upper 2–3 leaves are smaller, lanceolate or bract-like, and taper to a fine point. The bracts are green and usually tend toward violet at the edges, and they are lanceolate and taper to a fine point at the apex. The raceme is 5.5–15 cm long and ranges in color from pale pink to lilac (rarely white or bright magenta), and it is strongly scented with a flowering season from July to August. Inflorescence 11–26 cm, slender; peduncle with one to a few scattered, lanceolate bracts 1.5–6 cm; rachis 4–12 cm, densely many flowered; floral bracts lanceolate, often longer than ovary and flower, apex long acuminate-caudate. And flowers fragrant, pink, rarely pinkish white; ovary 5–8 mm including pedicel. Dorsal sepal broadly elliptic to broadly ovate-elliptic; lateral sepals reflexed, obliquely ovate, 4–5.5 × 3–4 mm, 3-veined, margin revolute, apex acute. Petals obliquely ovate-triangular, 3-veined, apex acute; lip spreading, broadly cuneate-obovate. There are two tubers with dimensions of 14–30 × 7.5–24 mm; the tubers are palmate lobed with thick segments that are tapering and obtuse, pressed together, and split halfway to the base into 3–6 lobes. The short and thick roots are sparse and grow horizontally or even toward the soil surface. The fruit has dimensions of 8.6–9.3 × 2.6–2.7 mm, and they are erect with six ribs. The seeds have dimensions of 0.3 × 0.1 mm and are produced in large numbers (http://www.theplantlist.org^1^; www.efloras.org^2^; Figure 1).

## Traditional uses

*G. conopsea* is widely distributed in northern Europe and certain Asian countries. Like some TCM, *G. conopsea* is not used in folk medicine, and just has been considered a fragrant orchid plant in some European countries. So, studies on the ethnopharmacology and clinical uses of the plant have been mainly focused on Asian countries, such as China, Nepal, and Japan.

In China, *G. conopsea* is primarily distributed in Heilongjiang, Jilin, Liaoning, Hebei, Shanxi, Shangxi, Gansu, Sichuan, and Yunnan Provinces and Inner Mongolia and Xizang Autonomous Regions at altitudes of 265–4700 m (Commission of Flora Reipublicae Popularis Sinicae, 2004). The tuber has been employed as a reinforcing agent of folk medicines and widely used as traditional Chinese medicine (Han national medicine), Tibetan medicine, Mongolian medicine, Baiyao (the Bai national medicine), Chaoyao (the Korean national medicine), and Naxiyao (the Naxi national medicine) to treat various diseases in China. The tuber has also been employed as a health care product with other medicines or food to improve the body and prevent illness, and could be made to tincture and galenical to treat impotence and the bronchial asthma in China and Russia, respectively (Mamedov and Craker, 2001; Matsuda et al., 2004; Gutierrez, 2010). But at the same time, because of the rare resource of this plant, the tuber of *Gymnadenia crassinervis, Coeloglossum viride* var. *bracteatum*, and *Spiranthea lancea* have been used as substitutes for *G. conopsea* in certain regions, such as Tibet region (Xie et al., 2005; Zi, 2008; Xue et al., 2009).

As a traditional Tibetan medicine, the *G. conopsea* tuber has been widely used in the Tibetan region to treat lung disease and weakening by invigorating the kidney and moisturizing the lungs. The plant is known as “Wangla,” and it has been recorded in the “Sibuyidian,” the classical book of Tibetan medicine, since the eighth century. In Tibetan medicine, it could be used as a single medicine or as one composition mixed with other medicines to treat diseases. For example, after grinding to powder, it (30 g) concoction with bee honey (40 g), *Rhizome Gastrodiae* (30 g), *Radix Phlomii* (30 g), *Herb Drosera peltat* (30 g), and *Rhododendron parvifolium* (30 g) could be used to treat impotence, spermatorrhea, anemia, and insomnia for 3 g twice per day (Chinese Materia Editorial Committee, State Chinese Medicine Administration Bureau, 2002). And according to the database of Tibetan prescriptions, out of 4500 traditional prescriptions in Tibet Autonomous Region, the *G. conopsea* tuber was used 104 times (rate of 2.3%), and 33 prescriptions were used to invigorate the body, strengthen the Yang, and lengthen human life; 26 prescriptions were used to treat kidney diseases; 12 prescriptions were used to treat gout and arthromyodynia diseases; 11 prescriptions were applied to treat lung disease; 7 prescriptions were used to treat eye diseases; and other prescriptions were used to treat parasitic diseases and additional diseases (Ji et al., 2009; Xue et al., 2009). Currently, the tuber of *G. conopsea* is combined with other medicines in various preparations to treat a number of diseases. Five preparations have been listed in the Chinese Pharmacopeia and approved by the State Food and Drug Administration of China of the People's Republic of China. Medicines such as “Shi Wei Shou Shen Powder” and “Fu Fang Shou Shen Wan” have been widely used to treat kidney asthenia, impotence and spermatorrhea, among other disorders (http://www.sfda.gov.cn, 2014; Table 1). Except the above effects on the clinic, the tuber of *G. conopsea* also could be used to treat hepatitis B by folk doctors only in the Tibetan region (Chinese Materia Editorial Committee, State Chinese Medicine Administration Bureau, 2002). At the same time, it has also been used as a common food item by local people in the Tibetan region, where it is usually cooked with vegetables and rice. For example, the tuber is used as an important ingredient in the famous dish “Shiguo Ji” (chicken cooked in a stone hotpot).

**Table 1:** Preparations in which *Gymnadenia conopsea* (L.) R. Br. was the primary component as listed in the Chinese Pharmacopeia and approved by the government^*^.

**Preparation name**	**Main compositions**	**Usage**
Shi Wei Shou Shen San	Tuber Gymnadenia, Cinnamomi Cortex, Myristicae Semen, Piperis Longi Fructus, Asparagi Radix, Granati Pericarpium, Canavaliae Semen, Carthami Flos, Moschus, Bear Gall.	Invigorating the kidney and treating spermatorrhea.
Fu Fang Shou Shen Wan	Tuber Gymnadenia, Polygonati Rhizoma, Cynomorii Herba, Chebulae Fructus, Rhizoma Mirabilis Himalaica, Asparagi Radix, Cordyceps, Tribuli Fructus, Rhizoma Przewalskia Tangutica, Herba Pleurospermum.	Warming the kidney and activating yang. Treating insufficiency of kidney-YANG, damage of essence, impotence, and spermatorrhea, etc.
Shou Shen Shen Bao Jiao Nang	Tuber Gymnadenia, Polygonati Rhizoma, Asparagi Radix, Folium Rhododendron Anthopogonoides, Cordyceps.	Warming the kidney and invigorating yin. Treating giddy dazzled, tinnitus, lumbar genu aching, and limp is faint induced by kidney asthenia.
Shou Zhang Shen 37 Wan	Tuber Gymnadenia, Calcite, Alpiniae Oxyphyliae Fructus, Granati Pericarpium, Zingiberis Rhizoma, Feces Trogopterori, Myristicae Semen, Piperis Longi Fructus, Gecko, Chebulae Fructus, Sal Ammoniac, Folium Rhododendron Anthopogonoides, etc.	Invigorating the kidney and strengthening yang. Treating kidney asthenia, edema, tinnitus, impotence, and spermatorrhea, etc.
Fu Fang Shou Shen Yi Zhi Jiao Nang	Tuber Gymnadenia, Polygoni Multiflori Radix Praeparata, Acanthopanacis Senticosi Radix, Polygonati Rhizoma, Angelicae Sinensis Radix, Asteragali Radix, Lycii Fructus, Schisandrae Chinensis Fructus, Corni Fructus, Rhizoma Polygala, Acori Tatarinowii Rhizoma, Paeoniae Radix Rubra.	Invigorating the kidney and liver, and treating the deficiency of liver and kidney, amnesia, palpitation, and insomnia induced by Qi and blood hemophthisis.

* *Cited from “Chinese Pharmacopeia” and the Website: http://www.sfda.gov.cn*.

In the traditional Mongolian medicine, the tuber of *G. conopsea* is named “Erihaoteng” and has been historically recorded by many classical folk medicine books of the Inner Mongolia Autonomous Region. In additional, it has been widely used to treat kidney asthenia, lumbago and leg pain, light scurvy, spermatorrhea, and impotence (Gege et al., 2013). The tuber of *G. conopsea* and other traditional medicines have been combined into different preparations to treat various diseases. For example, the tuber and an additional 36 medicines are known as Shouzhangsheng-37 pills, which are used to treat kidney cold and asthenia, edema, tinnitus, spermatorrhea, impotence, stomach diseases, dyspepsia, and other diseases, and this preparation has been approved by the State Administration of Traditional Chinese Medicine of the People's Republic of China (http://www.sfda.gov.cn, 2014; (Si and Liu, 2013); Table 1). Meanwhile, Wu et al. (2014) reported that in clinical practice of Mongolian medicine, after administrating orally two Shenzhujin pills (the tuber is the primary component) for three times per day for 21 days, the kidney deficiency of one patient (65 years) have been cured.

Furthermore, the tuber of *G. conopsea* is also used in Korean national medicine, Bai national medicine, and Naxi national medicine, where it is known as Yinyang Cao, Foshousheng, and Kaishelabei, respectively. However, it has been used as a reinforcing agent within all of these traditional medicines and used to treat similar diseases as previously indicated, such as invigorating the body, strengthening the Yang, etc. (Jia and Li, 2005). But up to now, except the experience-based uses of this plant, the relevant evidence-based clinical uses and the safety data scientifically are very rare and should be investigated further.

## Phytochemistry

**Figure 2:**
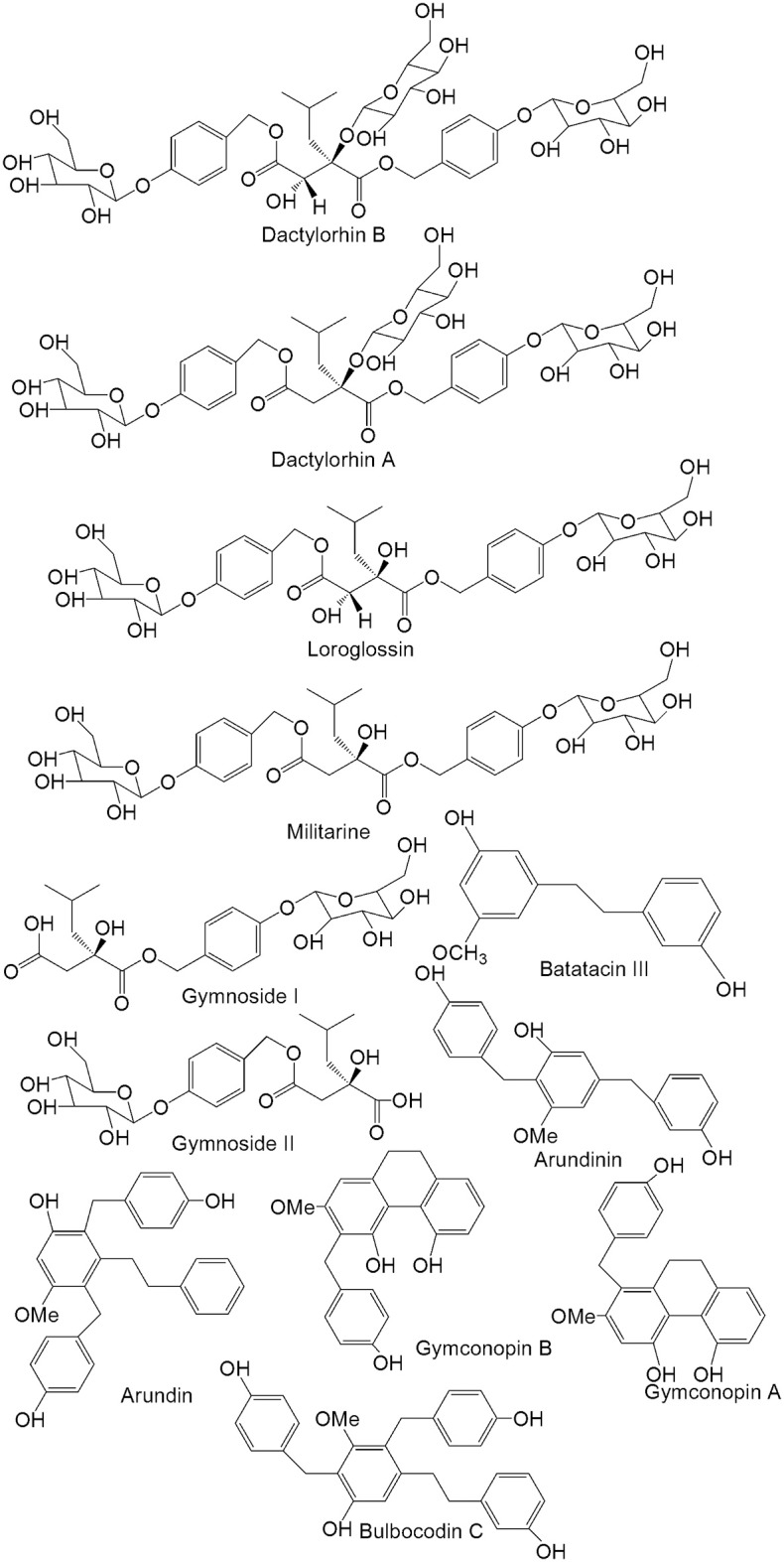
**Chemical structures of the primary compounds from the tuber of *Gymnadenia conopsea* (L.) R. Br**.

Approximately 129 compounds have been isolated and identified from *G. conopsea* to date, and most of compounds isolated from the tuber. Forty-nine glucosides compounds (one of the most important components) were identified, which could be divided into benzylester glucosides and other glucosides. Meanwhile, dihydrostilbenes, phenanthrenes, aromatic compounds, polysaccharides, and other compounds were also isolated and reported (Table 2; Figure 2). The different chemical compositions of *G. conopsea* provide the foundation for its different pharmacology activities.

**Table 2:** **Compounds isolated from *Gymnadenia conopsea* (L.) R. Br. (the structure of the primary compounds are illustrated in Figure 2)**.

**No**.	**Names**	**Parts**	**References**
1	Gymnoside I	Tuber	Morikawa et al., 2006a
2	Gymnoside II	Tuber	Morikawa et al., 2006a
3	Gymnoside III	Tuber	Morikawa et al., 2006a
4	Gymnoside IV	Tuber	Morikawa et al., 2006a
5	Gymnoside V	Tuber	Morikawa et al., 2006a
6	Gymnoside VI	Tuber	Morikawa et al., 2006a
7	Gymnoside VII	Tuber	Morikawa et al., 2006a
8	Gymnoside VIII	Tuber	Morikawa et al., 2006b
9	Gymnoside IX	Tuber	Morikawa et al., 2006b
10	Gymnoside X	Tuber	Morikawa et al., 2006b
11	Loroglossin	Tuber	Morikawa et al., 2006b
12	Dactylorhin A	Tuber	Morikawa et al., 2006b
13	Dactylorhin B	Tuber	Morikawa et al., 2006b
14	Militarine	Tuber	Morikawa et al., 2006b
15	(−)-4-[β-D-glucopyranosyl-(1→4)-β-D-glucopyranosyloxy] benzyl alcohol	Tuber	Zi, 2008; Zi et al., 2008
16	(+)-4-[α-D-glucopyranosyl-(1→4)-β-D-glucopyranosyloxy] benzyl alcohol	Tuber	Zi, 2008; Zi et al., 2008
17	(−)-4-[β-D-glucopyranosyl-(1→3)-β-D-glucopyranosyloxy] benzyl alcohol	Tuber	Zi, 2008; Zi et al., 2008
18	(−)-4-[β-D-glucopyranosyl-(1→3)-β-D-glucopyranosyloxy] benzyl ethyl ether	Tuber	Zi, 2008; Zi et al., 2008
19	(−)-(2R,3S)-1-(4-β-D-glucopyranosyloxybenzyl)-2-O-β-D-glucopyranosy]-4-{4-[α-D-glucopyranosyl-(1→4)-β-D-glucopyranosyloxy]benzyl}-2-isobutyltartrate	Tuber	Zi, 2008; Zi et al., 2008
20	(−)-(2R,3S)-1-(4-β-D-glucopyranosyloxybenzyl)-2-O-β-D-glucopyranosyl-4-{4-[β-D-glucopyranosyl-(1→3)-β-D-glucopyranosyloxy]benzyl}-2-isobutyltartrate	Tuber	Zi, 2008; Zi et al., 2008
21	(−)-(2R,3S)-1-{4-[β-D-glucopyranosyl-(1→3)-β-D-glucopyranosyloxy] benzyl}-2-O-β-D-glucopyranosyl-4-(4-β-D-glucopyranosyloxybenzyl)-2-isobutyltartrate	Tuber	Zi, 2008; Zi et al., 2008
22	(−)-(2R,3S)-1-(4-β-D-glucopyranosyloxybenzyl)-4-{4-[β-D-glucopyranosyl-(1→6)-β-D-glucopyranosyloxy]benzyl}-2-isobutyltartrate	Tuber	Zi, 2008; Zi et al., 2008
23	(−)-(2R,3S)-1-(4-β-D-glucopyranosyloxybenzyl)-4-methyl-2-isobutyltartrate	Tuber	Zi, 2008; Zi et al., 2008
24	(−)-(2R)-2-O-β-D-glucopyranosyl-4-(4-β-D-glucopyranosylbenzyl)-2-isobutyltartrate	Tuber	Zi, 2008; Zi et al., 2008
25	Dactylorhin E	Tuber	Zi, 2008
26	Coelovirins A	Tuber	Zi, 2008
27	Coelovirins B	Tuber	Zi, 2008
28	Coelovirins D	Tuber	Zi, 2008
29	Coelovirins E	Tuber	Zi, 2008
30	4-Hydroxybenzyl-β-D-glucopyranoside	Tuber	Yang, 2009
31	4-Methylphenyl-β-D-glucopyranoside	Tuber	Yang, 2009
32	4-Hydroxyphenyl-β-D-glucopyranoside	Tuber	Yang, 2009
33	4-Methoxymethylbenzyl-β-D-glucoside	Tuber	Morikawa et al., 2006b
34	bis (4-hydroxybenzyl)-ethermono-β-D-glucopyranoside	Tuber	Morikawa et al., 2006b
35	4-(β-D-glucopyranosyloxy) benzoic aldehyde	Tuber	Zi, 2008
36	4-(β-D-glucopyranosyloxy) benzyl ethyl ether	Tuber	Zi, 2008
37	Phenyl-β-D-glucopyranoside	Tuber	Morikawa et al., 2006b
38	4-Formylphenyl-β-D-glucopyranoside	Tuber	Morikawa et al., 2006b
39	Benzyl-β-D-glucopyranoside	Tuber	Morikawa et al., 2006b
40	trans-ferulic acid-β-D-glucoside	Tuber	Zi, 2008
41	cis-ferulic acid-β-D-glucoside	Tuber	Zi, 2008
42	N^6^-(4-hydroxybenzyl) adenine riboside	Tuber	Zi, 2008
43	Daucosterol	Tuber	Li et al., 2007
44	Dioscin	Tuber	Li et al., 2001
45	Dactylose A	Tuber	Morikawa et al., 2006b
46	Dactylose B	Tuber	Morikawa et al., 2006b
47	n-Butyl-β-D-fructopyranose	Tuber	Li et al., 2001
48	Thymidine	Tuber	Morikawa et al., 2006b
49	Batatasin III	Tuber	Matsuda et al., 2004
50	3′-O-methylbatatasin III	Tuber	Matsuda et al., 2004
51	3′,5-Dihydroxy-2-(4-hydroxybenzyl)-3-methoxybibenzyl	Tuber	Matsuda et al., 2004
52	3,3′-Dihydroxy-2-(4-hydroxybenzyl)-5-methoxybibenzyl	Tuber	Matsuda et al., 2004
53	Gymconopin D	Tuber	Matsuda et al., 2004
54	3,3′-Dihydroxy-2,6-bis(4-hydroxybenzyl)-5-methoxybibenzyl	Tuber	Matsuda et al., 2004
55	5-O-methylbatatacin III	Tuber	Yoshikawa et al., 2005
56	2-(4-Hydroxybenzyl)-3′-O-methylbatatacin III	Tuber	Yoshikawa et al., 2005
57	Arundinin	Tuber	Yoshikawa et al., 2005
58	Arundin	Tuber	Yoshikawa et al., 2005
59	Bulbocodin C	Tuber	Yoshikawa et al., 2005
60	Bulbocodin D	Tuber	Yoshikawa et al., 2005
61	Gymconopin A	Tuber	Matsuda et al., 2004
62	Gymconopin B	Tuber	Yoshikawa et al., 2005
63	Gymconopin C	Tuber	Yoshikawa et al., 2005
64	1-(4-Hydroxybenzyl)-4-methoxy-9,10-dihydrophenanthrene-2,7-diol	Tuber	Matsuda et al., 2004
65	1-(4-Hydroxybenzyl)-4-methoxyphenanthrene-2,7-diol	Tuber	Matsuda et al., 2004
66	Dihydrophenanthrene-4,5-diol	Tuber	Matsuda et al., 2004
67	2-Methoxy-9,10-4-methoxy-9,10-dihydrophenanthrene-2,7-diol	Tuber	Matsuda et al., 2004
68	Blestriarene A	Tuber	Matsuda et al., 2004
69	Blestriarene A	Tuber	Matsuda et al., 2004
70	Blestriarene A	Tuber	Matsuda et al., 2004
71	9, 10-Dihydro blestriarene B	Tuber	Matsuda et al., 2004
72	9,10–9′,10′-Dihydro blestriarene C	Tuber	Matsuda et al., 2004
73	4-Hydroxybenzyl alcohol	Tuber	Cai et al., 2006
74	4-Hydroxybenzyl aldehyde	Tuber	Cai et al., 2006
75	Pinoresinol	Tuber	Morikawa et al., 2006b
76	4-Hydroxybenzoic acid	Tuber	Yue et al., 2010
77	Vanillic acid	Tuber	Yue et al., 2010
78	Trans-p-coumaric acid	Tuber	Yue et al., 2010
79	Cis-p-coumaric acid	Tuber	Yue et al., 2010
80	4-Benzaldehyde	Tuber	Morikawa et al., 2006b
81	4-Hydroxybenzyl methyl ether	Tuber	Zi, 2008
82	3,5-Methoxy benzaldehyde	Tuber	Morikawa et al., 2006b
83	4-[(4-Hydroxyphenyl)methoxy]-benzenemethanol	Tuber	Morikawa et al., 2006b
84	4,4′-Dihydroxydiphenyl methane	Tuber	Morikawa et al., 2006b
85	Phenol	Tuber	Morikawa et al., 2006b
86	5-Hydroxymethyl furfural	Tuber	Morikawa et al., 2006b
87	1,2-Dihydroxy benzene	Tuber	Morikawa et al., 2006b
88	2,6-Dimethoxy phenol	Tuber	Morikawa et al., 2006b
89	Eugenol	Tuber	Morikawa et al., 2006b
90	4-Hydroxybenzene	Tuber	Morikawa et al., 2006b
91	4-Methoxy phenylpropanol	Tuber	Morikawa et al., 2006b
92	4-Ethoxy phenylpropanol	Tuber	Morikawa et al., 2006b
93	Contra-hydroxybenzyl dithioether	Tuber	Morikawa et al., 2006b
94	Syringol	Tuber	Morikawa et al., 2006b
95	Syringaldehyde	Tuber	Morikawa et al., 2006b
96	Gastrodin	Tuber	Yue et al., 2010
97	4-Hydroxybenzoic aldehydes	Tuber	Zi, 2008
98	4-Hydroxybenzoic acid	Tuber	Yue et al., 2010
99	3-Hydroxybenzoic acid	Tuber	Yue et al., 2010
100	4-Hydroxyisophthalic acid	Tuber	Yue et al., 2010
101	3-Methoxy-4-hydroxybenzoic acid	Tuber	Yue et al., 2010
102	Arctigenin	Tuber	Yue et al., 2010
103	erythro-buddlenol E	Tuber	Zi, 2008
104	Lappaol A	Tuber	Yue et al., 2010
105	Lappaol F	Tuber	Yue et al., 2010
106	2-Hydroxy-2-(4-hydroxyphenylmethyl)-4-methylcyclopent-4-en-1,3-dione	Tuber	Zi, 2008
107	2-Hydroxy-3-(4-hydroxyphenyl)-4-hydroxymethylcyclopent-2-enone	Tuber	Zi, 2008
108	3,3′-Dihydroxy-4-(4-hydroxybenzyl)-5-methoxybibenzyl	Tuber	Morikawa et al., 2006b
109	2-C-(4-hydroxybenzyl)-α-L-xylo-3-ketohexulofuranosono-1,4-lactone	Tuber	Morikawa et al., 2006b
110	Arabinose	Tuber	Sun et al., 2010
111	Xylose	Tuber	Sun et al., 2010
112	Mannitose	Tuber	Sun et al., 2010
113	Galactose	Tuber	Sun et al., 2010
114	Glucose	Tuber	Sun et al., 2010
115	Secoxyloganin	Tuber	Zi, 2008
116	Quercitin-3, 7-di-O-β-D-glucopyranoside	Tuber	Zi, 2008
117	Kaempferol-3-β-glycosido-7-β-glycoside	Flower	Schonsiegel and Ingomar, 1969
118	Quercetin-3-β-glycosido-7-glycoside	Flower	Schonsiegel and Ingomar, 1969
119	Astragalin	Flower	Schonsiegel and Ingomar, 1969
120	Isoquercetin	Flower	Schonsiegel and Ingomar, 1969
121	Cyclo(L-Leu-L-Tyr)	Tuber	Zi, 2008
122	Cyclo(L-Leu-L-Pro)	Tuber	Zi, 2008
123	Cyclo(L-Val-L-Tyr)	Tuber	Zi, 2008
124	Cyclo(L-Ala-L-Phe)	Tuber	Zi, 2008
125	Thymidine	Tuber	Yue et al., 2010
126	β-sitoterol	Tuber	Yue et al., 2010
127	Octadecylselyl acid	Tuber	Yue et al., 2010
128	Di-(p-hydroxybenzyl) disulfide	Tuber	Li et al., 2007
129	4.4′-Dihydroxybenzyl sulfoxide	Tuber	Li et al., 2007

### Glucosides

As one of the most important components, glucosides are widely studied and separated from the tuber of *G. conopsea*. According to the structural conformation of the glucosides, they have been divided into benzylester glucosides and other glucosides.

### Benzylester glucosides

Benzylester glucosides are produced by combining 2-isobutyl tartaric acid or 2-isobutyl hydroxysuccinic acid with 4-glycosylbenzyl alcohol. According to the differences in their organic acids, they are classified as (2R, 3S)-2-isobutyl tartaric acid derivates and (2R)-2-isobutyl hydroxysuccinic acid derivatives. Approximately 29 compounds have been isolated and identified. In 2006, Morikawa et al. (2006a,b) isolated the following compounds: gymnoside I (**1**), gymnoside II (**2**), gymnoside III (**3**), gymnoside IV (**4**), gymnoside V (**5**), gymnoside VI (**6**), gymnoside VII (**7**), gymnoside VIII (**8**), gymnoside IX (**9**), gymnoside X (**10**), loroglossin (**11**), dactylorhin A (**12**), dactylorhin **B** (**13**)and militarine (**14**). Meanwhile, (−)-4-[β-D-glucopyranosyl-(1→4)-β-D-glucopyranosyloxy] benzyl alcohol (**15**), (+)-4-[α-D-glucopyranosyl-(1→4)-β-D-glucopyranosyloxy] benzyl alcohol (**16**), (−)-4-[β-D-glucopyranosyl-(1→3)-β-D-glucopyranosyloxy] benzyl alcohol (**17**), (−)-4-[β-D-glucopyranosyl-(1→3)-β-D-glucopyranosyloxy] benzyl ethyl ether (**18**), (−)-(2R,3S)-1-(4-β-D-glucopyranosyloxybenzyl)-2-O-β-D-glucopyranosy]-4-{4-[α-D-glucopyranosyl-(1→4)-β-D-glucopyranosyloxy]benzyl}-2-isobutyltartrate (**19**), (−)-(2R,3S)-1-(4-β-D-glucopyranosyloxybenzyl)-2-O-β-D-glucopyranosyl-4-{4-[β-D-glucopyranosyl-(1→3)-β-D-glucopyranosyloxy] benzyl}-2-isobutyltartrate (**20**), (−)-(2R,3S)-1-{4-[β-D-glucopyranosyl-(1→3)-β-D-glucopyranosyloxy]benzyl}-2-O-β-D-glucopyranosyl-4-(4-β-D-glucopyranosyloxybenzyl)-2-isobutyl-tartrate (**21**), (−)-(2R,3S)-1-(4-β-D-glucopyranosyloxybenzyl)-4-{4-[β-D-glucopyranosyl-(1→6)-β-D-glucopyranosyloxy]benzyl}-2-isobutyltartrate (**22**), (−)-(2R,3S)-1-(4-β-D-glucopyranosyloxybenzyl)-4-methyl-2-isobutyltartrate (**23**) and (−)-(2R)-2-O-β-D-glucopyranosyl-4-(4-β-D-glucopyranosylbenzyl)-2-isobutyltartrate (**24**) were isolated from the tuber in 2008. And the further studies showed that at 10^−5^ M, the inhibition rates of the above compounds foracetylcholine esterase were <10%, and the positive drug donepezil gave an inhibition rate of 77.2%. Then, the results presented that at 10^−5^ M, the inhibition rates of the above compounds formonoamine oxidase-B were <15.2%, and the positive drug pargyline had an inhibition rate of 94.5% (Zi et al., 2008). Meanwhile, dactylorhin E (**25**), coelovirins A (**26**), coelovirins B (**27**), coelovirins D (**28**), and coelovirins E (**29**) also were isolated (Zi, 2008).

### Other glucosides

Other glucosides have also been isolated from the tuber of *G. conopsea*. In 2009, Yang identified 4-hydroxybenzyl-β-D-glucopyranoside (**30**), 4-methylphenyl-β-D-glucopyranoside (**31**), and 4-hydroxyphenyl-β-D-glucopyranoside (**32**). The following compounds have also been isolated (Li et al., 2001; Morikawa et al., 2006b; Zi, 2008): 4-methoxymethylbenzyl-β-D-glucoside (**33**), *bis* (4-hydroxybenzyl)-ether mono-β-D-glucopyranoside (**34**), 4-(β-D-glucopyranosyloxy) benzoic aldehyde (**35**), 4-(β-D-glucopyranosyloxy)benzyl ethyl ether (**36**), phenyl-β-D-glucopyranoside (**37**), 4-formylphenyl-β-D-glucopyranoside (**38**), benzyl-β-D-glucopyranoside (**39**), *trans*-ferulic acid-β-D-glucoside (**40**), *cis*-ferulic acid-β-D-glucoside (**41**), N^6^-(4-hydroxybenzyl)adenine riboside (**42**), daucosterol (**43**), dioscin (**44**), dactylose A (**45**), dactylose B (**46**), n-butyl-β-D-fructopyranose (**47**), and thymidine (**48**).

### Dihydrostilbenes

Dihydrostilbenes are other important components of *G. conopsea*. The basic parental nucleus of these compounds is dihydrogen alkene. The C-2, -3, -4, -5, -6 and -3′ positions of the basic nucleus areusually substituted. In 2004 and 2005, batatacin III (**49**), 3′-O-methylbatatacin III (**50**), 3′,5-dihydroxy-2-(4-hydroxybenzyl)-3-methoxybibenzyl (**51**), 3,3′-dihydroxy-2-(4-hydroxybenzyl)-5-methoxybibenzyl (**52**), gymconopin D (**53**), 3,3′-dihydroxy-2,6-bis(4-hydroxybenzyl)-5-methoxybibenzyl (**54**), 5-O-methylbatatacin III (**55**), 2-(4-hydroxybenzyl)-3′-O-methylbatatacin III (**56**), arundinin (**57**), arundin (**58**), bulbocodin C (**59**), bulbocodin D (**60**) were isolated and identified from the tuber, respectively (Matsuda et al., 2004; Yoshikawa et al., 2005).

### Phenanthrenes

A group of phenanthrene compounds was isolated from the tuber of *G. conopsea* and studied in recent decades, including gymconopin A (**61**), gymconopin B (**62**), gymconopin C (**63**), 1-(4-hydroxybenzyl)-4-methoxy-9,10-dihydrophenanthrene-2,7-diol (**64**), 1-(4-hydroxybenzyl)-4-methoxyphenanthrene-2,7-diol (**65**), 2-methoxy-9,10-dihydrophenanthrene-4,5-diol (**66**), 4-methoxy-9,10-dihydrophenanthrene-2,7-diol (**67**), blestriarene A (**68**), blestriarene A (**69**), blestriarene A (**70**), 9, 10-dihydro blestriarene B (**71**), and 9,10–9′,10′-dihydro blestriarene C (**72**) (Matsuda et al., 2004; Yoshikawa et al., 2005).

### Aromatic compounds

Approximately 20 aromatic compounds from *G. conopsea* have been studied, and most of them are phenol compounds. In 2006, Cai et al. (2006) isolated 4-hydroxybenzyl alcohol (**73**) and 4-hydroxybenzyl aldehyde (**74**) from the tuber. Then, pinoresinol (**75**), 4-hydroxybenzoic acid (**76**), vanillic acid (**77**), *trans*-p-coumaric acid (**78**), *cis*-p-coumaric acid (**79**), 4-benzaldehyde (**80**), 4-hydroxybenzyl methyl ether (**81**), 3,5-methoxy benzaldehyde (**82**), 4-[(4-hydroxyphenyl)methoxy]-benzenemethanol (**83**), 4,4′-dihydroxydiphenyl methane (**84**), phenol (**85**), 5-hydroxymethyl furfural (**86**), 1,2-dihydroxybenzene (**87**), 2,6-dimethoxy phenol (**88**), eugenol (**89**), 4-hydroxybenzene (**90**), 4-methoxy phenylpropanol (**91**), 4-ethoxy phenylpropanol (**92**), contra-hydroxybenzyl dithioether (**93**), syringol (**94**), syringaldehyde (**95**), gastrodin (**96**), 4-hydroxybenzoic aldehydes (**97**), 4-hydroxybenzoic acid (**98**), 3-hydroxybenzoic acid (**99**), 4-hydroxyisophthalic acid (**100**), 3-methoxy-4-hydroxybenzoic acid (**101**), arctigenin (**102**), erythro-buddlenol E (**103**), lappaol A (**104**), lappaol F (**105**), 2-hydroxy-2-(4-hydroxyphenylmethyl)-4-methylcyclopent-4-en-1,3-dione (**106**), 2-hydroxy-3-(4-hydroxyphenyl)-4-hydroxymethylcyclopent-2-enone (**107**), 3,3′-dihydroxy-4-(4-hydroxybenzyl)-5-methoxybibenzyl (**108**), and 2-c-(4-hydroxybenzyl)-α-L-xylo-3-ketohexulofuranosono-1,4-lactone (**109**) were isolated and identified from the tuber of *G. conopsea* (Morikawa et al., 2006b; Zi, 2008; Yue et al., 2010).

### Polysaccharides

In 2009, Yang (2009) studied the four extraction and purification methods for the polysaccharides of *G. conopsea*. The results showed that the optimum parameters for solvent extraction were 80°C for 2.5 h, a ratio of material to water of 1:30 with three repetitions, which yielded a polysaccharide extraction rate of up to 11.83%. The microwave extraction method were 250 W for 20 min, a ratio of material to water of 1:30 with one repetition, and the extraction rate was up to 13.56%. The ultrasound extraction method was 240 W, 60°C for 10 min, and three repetitions, and the extraction rate was 15.24%. Finally, the parameters for cellulose extraction were to add cellulose at 1% of the tuber concentration for 4 h at 50°C and Ph 4.8, and the extraction rate was 16.58%. Thus, the cellulose method is the best among the four methods. Meanwhile, vapor phase chromatography was adopted to analyze the monosaccharides from the polysaccharides, and the results showed that the polysaccharides are composed of arabinose (**110**), xylose (**111**), mannitose (**112**), galactose (**113**), and glucose (**114**) (Sun et al., 2010). And in 2014, the water soluble polysaccharides from the tuber of *G. conopsea* collected at seven regions in China were investigated and compared using high performance size exclusion chromatography coupled with multi-angle laser light scattering/refractive index detector (HPSEC-MALLS/RID) and saccharide mapping based on polysaccharide analysis by carbohydrate gel electrophoresis (PACE), respectively. The results showed that the weight-average molecular weight (M_w_) and the radius of gyration (< S^2^>z^1/2^) of polysaccharides were ranging from 4.46 × 105 to 7.41 × 105 Da and 73.3–94.2 nm, respectively. And the exponent (*v*) values of < S^2^>z^1/2^ = ${\text{kM}}_{\text{w}}^{\text{v}}$ were ranging from 0.36 to 0.42, which indicated that α-1,4-and β-1,3(4)-glucosidic, α-1,5-arabinosidic, β-1,4-mannosidic and α-1,4-D-galactosiduronic linkages existed in globular polysaccharides. The further studies results that the nitric oxide released from RAW 264.7 cells induced by polysaccharides were significantly affected by their α-1,5-arabinosidic and β-1,3(4)-glucosidic, especially α-1,4-D-galactosiduronic and β-1,4-mannosidic linkages (Lin et al., 2015).

### Other compounds

From *G. conopsea*, six flavones were isolated from the flower, including secoxyloganin (**115**), quercitin-3, 7-di-O-β-D-glucopyranoside (**116**), kaempferol-3-β-glycosido-7-β-glycoside (**117**), and quercetin-3-β-glycoside-7-glycoside (**118**), astragalin (**119**) and isoquercetin (**120**) (Schonsiegel and Ingomar, 1969; Zi, 2008). Then, cyclo(L-Leu-L-Tyr) (**121**), cyclo(L-Leu-L-Pro) (**122**), cyclo(L-Val-L-Tyr) (**123**), cyclo(L-Ala-L-Phe) (**124**), thymidine (**125**), β-sitoterol (**126**), and octadecylselyl acid (**127**) were isolated (Yue et al., 2010; Zi et al., 2010). And di-(p-hydroxybenzyl) disulfide (**128**) and 4.4′-dihydroxybenzyl sulfoxide (**129**) also were identified (Li et al., 2007). Lan (2005) determined the trace element contents of the tuber of *Gymnadenia conopsea* (L.) R. Br., and the results showed that the Cu, Mn, Fe, Zn, Ni, Se, Cr, K, Ca, Mg, Cd, and Pb contents in μg/g were 7.6, 31.6, 60.3, 20.1, 2.1, 6.0, 2.0, 19,000, 4100, 1800, 0.16, and 3.31, respectively.

## Pharmacology

### Tonifying effect and anti-fatigue activity

In China, the *G. conopsea* tuber has been used as a reinforcing agent for traditional medicines, and it has historically been used to treat kidney asthenia, lung asthenia, consumption diseases, neurasthenia, impotence, spermatorrhea, and other diseases. According to the theory of traditional Chinese medicine, kidney asthenia is related to weakness in the body. Thus, the tonifying activity (kidney-yang-tonifying activity) was the primary subject of study in China. The results showed that after administering orally 2 and 1 g/kg the tuber for 10 days, the medicine relieved the symptoms in mice with yang deficiencies that had been induced by hydrocortisone (25 mg/kg, i.g.), increased the body weight of kidney-yang-deficient mice (7.82 and 5.56 g, *P* > 0.05), prolonged the retention time on the rotating bar (19.57 ± 10.21 min, *P* < 0.01, and 5.90 ± 2.47 min, *P* < 0.05) and increased the kidney indexes of treated mice compared with those of the model group. And the increased weight and time on the rotating bar for the untreated control groups were 8.71 g (*P* > 0.05) and 24.20 ± 13.95 min (*P* < 0.01), respectively. Further, study showed that the tubers could also increase DNA synthesis in the spleen (48.89 ± 18.68 and 46.63 ± 13.80 μg/g, *P* < 0.01), kidneys (14.26 ± 5.34 and 5.94 ± 4.88 μg/g, *P* > 0.05), and liver (42.94 ± 13.95 μg/g and 42.02 ± 17.69, *P* < 0.05) of mice with yang deficiencies that were induced by hydrocortisone compared with the mice in the model group (27.25 ± 13.18 μg/g, *P* < 0.01, 4.90 ± 4.82 μg/g, *P* > 0.05, and 19.02 ± 15.49 μg/g). These results suggested that the tuber has a significant tonifying effect on the kidney and strengthens the bodies of mice with kidney deficiencies that were induced by hydrocortisone (Lin, 2009).

In 2011, Zhao and Liu (2011) studied the anti-fatigue activity of the tuber of *G. conopsea* in mice. After administering orally (i.g.) aqueous extracts to mice (40, 20, and 10 g/kg) for 6 days, the swimming times were markedly increased (1441.6, 1357.0, and 1249.9 s, *P* < 0.01) during the weight-bearing swimming tests in a dose-dependent manner. The swimming times of the control and positive groups (*Radix ginseng*, 20 g/kg) were 491.1 s and 1685.9 s (*P* < 0.01). According to the processing method of traditional Mongolia medicine in China, the tuber should be decocted in goat or cow milk before clinical use. After processing with a different method, Jin and Wang (2009) studied and compared the strengthening effects of the tuber of *G. conopsea* tonics. The results showed that compared with the control group (saline water, 6.38 ± 4.22 min), a goat milk decoction (2 g/kg. i.g.) could significantly increase the swimming time of mice (19.44 ± 12.6 min, *P* < 0.01); the unprocessed group (2 g/kg) and cow's milk decoction group (2 g/kg) had swimming times of 9.09 ± 9.81 min and 6.43 ± 7.21 min (*P* > 0.05), respectively, but they did not show significant difference. All of the decoctions could increase the SOD activity and MDA content compared with that of the control group. Thus, the researchers indicated that after processing with goat milk, the tuber have a better effect with the strengthening tonics than unprocessed medicine (**Table 4**).

### Anti-oxidant activity

The anti-oxidant activity of the tuber of *G. conopsea* was first comprehensive studied in 2006. The results showed that after isolating by Diaion HP-20 column chromatography (reverse-phase silica gel columnchromatography, Chromatorex ODSDM1020T, 100–200 mesh, Fuji Silysia Chemical, Ltd.), the MeOH-eluted fraction showed the radical scavenging activities for DPPH radical (SC_50_ = 59.1 μg/ml) and.${\text{O}}_{2}^{-}$ (IC_50_ = 13.3 μg/ml), but without the inhibitory activity on xanthine oxidase (IC_50_ > 100 μg/ml). Meanwhile, the radical scavenging activities for DPPH radical and^.^${\text{O}}_{2}^{-}$ exhibiting inhibitory activity on formozan formation and xanthine oxidase of the acetone-eluted fraction were 55.4, 33.2, and 29.7 μg/ml, respectively. But H_2_O-eluted fraction didn't present any activities. Then, the anti-oxidant 13 compounds were studied further, some compounds showed the marked radical scavenging activities for DPPH radical and^.^${\text{O}}_{2}^{-}$ exhibiting inhibitory activity on formozan formation and xanthine oxidase of 13 compounds (Morikawa et al., 2006b; Table 3). Meanwhile, the study simultaneously showed that at a concentration of 10^−6^ mol/L, arctigenin, lappaol A, and lappaol F have anti-oxidative activities that inhibit Fe^2+^-cystine-induced lipid peroxidation in rat liver microsomes with inhibitory rates of 53, 59, and 52%, respectively, relative to that of vitamin E at 35% (Zi et al., 2008).

**Table 3:** **Activities of specific compounds from *Gymnadenia conopsea* (L.) R. Br**.

**Compounds**	**Effects**	***In vitro* test**	**References**
Gymconopin A	Anti-oxidant activity	Radical scavenging activities for DPPH radical and.${\text{O}}_{2}^{-}$ exhibiting inhibitory activity on formozan formation and xanthine oxidase at 29.2, 45.8, and >100 μM; 33.4, 21.5, and >100 μM; 31.2 μM, >100 μM and null; 12.7, 0.95, and 44.0 μM; 8.2, 0.19, and 30.5 μM; 15.7, 9.4, and >100 μM; 5.8, 0.27, and 4.5 μM; >40, 82.8, and >100 μM; >40, 9.3, and 72.9 μM; >40, 13.4, and 45.1 μM; >40, 13.4, and 65.2 μM; 11.0, null and null; 6.0, 1.5, and >10 μM, respectively.	Morikawa et al., 2006b
GymconopinB			
2-Methoxy-9,10-dihydrophenanthrene-4,5-diol			
4-Methoxy-9,10-dihydrophenanthrene-2,7-diol			
1-(4-Hydroxybenzyl)-4-methoxy-9,10-dihydrophenanthrene-2,7-diol			
1-(4-Hydroxybenzyl)-4-methoxyphenanthrene-2,7-diol			
Blestriarene A			
BatatacinIII 3′,5-Dihydroxy-2-(4-hydroxybenzyl)-3-methoxybibenzyl3,3′-Dihydroxy-2-(4-hydroxybenzyl)-5-methoxybibenzyl			
3,3′-Dihydroxy-2,6-bis-(4-hydroxybenzyl)-5-methoxybibenzyl			
α-Tocopherol			
(+)-Catechin			
Arctigenin Lappaol A Lappaol F	Anti-oxidant activity	Anti-oxidative activity inhibiting Fe^2+^-cystine activity induced in rat liver microsomal lipid peroxidation with inhibitory rates of 53, 59, and 52%, compared with that of vitamin E at 35%.	Zi et al., 2008
(−)-4-[β-D-glucopyranosyl-(1→4)-β-D-glucopyranosyloxy]benzyl alcohol(−)-4-[β-D-glucopyranosyl-(1→3)-β-D-glucopyranosyloxy] benzyl ethyl ether(−)-(2R,3S)-1-[4-β-D-glucopyranosyloxybenzyl]-4-methyl-2-isobutyltartrate	Anti-HIV activity	At a concentration of 10^−5^ mol/L, the inhibition rates of these compounds against VSVC/HIV-luc model in 293 cell lines were 9.0, 5.0, 6.2, 11.9, 11.3, 0.6, 13.3, 5.1, 10.0, 2.4, and 0.6%, respectively. Comparisons with the positive drugs zidovudine (10^−7^ mol/L, 85.6%) and lamivudine (10^−8^ mol/L, 47.4%) were performed.	Zi, 2008
Cyclo[gly-L-S-(4-hydroxybenzyl)]cys 2-Hydroxy-2-(4-hydroxyphenylmethyl)-4-methylcyclopent-4-en-1,3-dione 2-Hydroxy-3-(4-hydroxyphenyl)-4-hydroxymethylcyclopent-2-enone			
Coelovirins E			
Dactylorhin E			
Dactylorhin B			
Militarine			
Gastrodin			

In 2010, Li et al. (2010) screened and evaluated the aqueous extract, acidic aqueous extract (pH = 3), 60% ethanol extract, 95% ethanol extract, n-butanol extract, n-butanol saturated by aqueous extract and ethyl acetate extracts of the tuber of *G. conopsea* for their radical scavenging capacity. The results showed that the radical scavenging capacity of the aqueous and acidic aqueous (pH = 3) extracts were weak. The radical scavenging activities (IC_50_) of the other extracts were 0.1266, 0.4537, 0.3151, and 0.1305 mg /ml. Thus, the investigators indicated that the 95% ethanol extract was the most effective among the six extracts (Table 4).

**Table 4:** **The biological activities of *Gymnadenia conopsea* (L.) R. Br**.

**Pharmacological activities**	**Plant parts**	**Processing method/extract**	**Test system**	**Animal or test organism**	**Positive drug**	**References**
Tonifying activity	Tuber	Powder, no extract	*In vivo*	Mice induced by hydrocortisone (25 mg/kg, i.g.)	–	Lin, 2009
Anti-fatigue activity	Tuber	Aqueous extract	*In vivo*	Mice	Radix ginseng, 20 g/kg	Zhao and Liu, 2011
	Tuber	Goat and cow milk decoction	*In vivo*	Mice	–	Jin and Wang, 2009
Anti-oxidant activity	Tuber	Methanol, aqueous and acetone-eluted fraction	*In vitro*	Radical scavenging assay	–	Morikawa et al., 2006b
	Tuber	Aqueous, acidic aqueous, 60% ethanol t, 95% ethanol, n-butanol, and n-butanol extracts	*In vitro*	Radical scavenging assay	–	Li et al., 2010
Anti-viral activity	Tuber	Aqueous extract	*In vitro*	The patient's serum with hepatitis B	–	Lu et al., 2002
Sedative and hypnotic activities	Tuber	Powder, no extract	*In vivo*	Mice	Diazepam	Lin, 2009
Preventing and treating gastric ulcers	Tuber	Powder, no extract	*In vivo*	Mice induced by a hydrochloride-ethanol solution	–	Lin, 2009
	Tuber	Powder, no extract	*In vivo*	Mice induced by a hydrochloride-ethanol solution	–	Jiang et al., 2009
Immunoregulatory Activity	Tuber	The polysaccharide	*In vivo*	Mice	–	Shang et al., 2014
Anti-aging activity	Tuber	Shouzhangshen-37 pill (the tuber of *G. conopsea* is the primary component of this pill)	*In vivo*	Mice induced by injecting D-galactose	Vitamin E	Si and Liu, 2013
Anti-hyperlipidemia activity	Tuber	Ethanol extracts	*In vivo*	Hyperlipidemia rats	Lovastatin	Zhang et al., 2013
Antianaphylaxis activity	Tuber	Methanolic extract	*In vivo*	Mice	–	Matsuda et al., 2004
Anti-silicosis activity	Tuber	60% ethanol extract	*In vivo*	Rat lung interstitial fibrosis	–	Wang et al., 2007, 2008
	Tuber	Ethanol extract	*In vivo*	Rat lung interstitial fibrosis	–	Zeng et al., 2007
	Tuber	Ethanol extract	*In vivo*	Rat lung interstitial fibrosis	–	Chen, 2008

### Anti-viral activity

In 2008, Zi (2008) studied the anti-HIV activity of certain compounds from the tuber of *G. conopsea* at a concentration of 10^−5^ mol/L in relation to the drugs zidovudine (10^−7^ mol/L, 85.6%) and lamivudine (10^−8^ mol/L, 47.4%), and the inhibition rates of these compounds against the VSVC/HIV-luc model in 293 cell lines were 0.6–13.3% (Table 3).

In Tibet, *G. conopsea* has primarily been used by local people to treat chronic hepatitis B. In 2002, Lu et al. (2002) studied the anti-HBV activity. The results showed that after treating the patient's serum that contained hepatitis B for 4 h, the medicine (0.03, 0.06, and 0.12 mg/L) could inhibit eight hemagglutin units of HBsAg. Thus, they indicated that the tuber has medium anti-HBV activity. In 2003, Kimura et al. (2003) studied the inhibitory effect of Tibetan medicinal plants on viral polymerases. The results showed that at 100 μg/ml, 28 species of 76 medicines presented the anti-RTase activity with the more than 70% inhibition rates. But after adding BSA to these drugs, except the fruit of *Terminalia chebula* and *Areca catechu*, other species didn't show the anti-RTase activity, including *G. conopsea* (Kimura et al., 2003; Table 4).

### Sedative and hypnotic activities

In 2009, Lin (2009) studied the sedative and hypnotic effects of the tuber of *Gymnadeniae* tubers at different dosages. In the sedative test, administering the tuber (2 and 1 g/kg i.g.) for 30 min, it could reduce the spontaneous activity of mice within 5 min (6.63 ± 3.18 and 5.36 ± 3.44) at inhibitory rates of 37.44% (*P* < 0.05) and 45.60% (*P* < 0.05), and the control group (normal saline) and positive group (diazepam, 0.004 g/kg) exhibited inhibition of 10.60 ± 5.77 and 3.66 ± 1.47 (an inhibition of 64.21%, *P* < 0.01), respectively. The tubers could also decrease the frequency of upward-raising motion in mice forelimbs within 5 min, especially for the high dose group at 15.42 ± 11.77 times (*P* < 0.05). The upward-raising frequency of the control and positive groups were 22.11 ± 6.92 and 9.37 ± 3.65 (*P* < 0.01), respectively.

In the mesmerism test, the medicine could prolong sleeping times at doses of 2 and 1 g/kg i.g. (32.94 ± 14.84 min, *P* < 0.01; and 28.85 ± 11.28 min, *P* < 0.05, respectively) in mice and decrease the latency time (3.00 ± 0.82 min, and 3.34 ± 0.52 min, *P* > 0.05, respectively) induced by pentobarbital sodium over the threshold dose (50 mg/kg) compared with mice in the control group (13.96 ± 8.45 and 4.80 ± 1.85 min); the positive groups were 45.31 ± 12.31 min (*P* < 0.01) and 1.05 ± 0.31 min (*P* < 0.05). This treatment also increased the number of sleeping mice (18 and 18) and latency time (3.11 ± 1.00 min, *P* < 0.01; and 2.56 ± 0.49 min, *P* < 0.05), and it prolonged their sleeping times (28.22 ± 10.50 min, *P* < 0.01; and 20.81 ± 9.22 min, *P* < 0.05) relative to that of the control group (7, 0.92 ± 1.58 and 8.56 ± 15.53 min), which was induced by pentobarbital sodium at less than the threshold dose (45 mg/kg), and the diazepam group (20, 6.35 ± 0.82 and 38.22 ± 3.84; *P* < 0.01). These results indicated that the tuber of *G. conopsea* has marked sedative and hypnotic dose-dependent effects, and its mechanism of action requires further research (Table 4).

### Preventing and treating gastric ulcers

With normal saline as the control, the inhibitory effect of the tubers on gastric ulcers was induced by hydrochloride-ethanol solution (7.5 ml/kg for 3 days, i.g.). The macroscopic and pathological results showed that at doses of 2 and 1 g/kg, the treatment could relieve the pathology index of gastric ulcers induced by hydrochloride-ethanol solution and decrease the gastric ulcer index (0.05 ± 0.13 cm and 0.22 ± 0.38 cm) at inhibitions of 88.86% (*P* < 0.01) and 48.88%, respectively. The biochemical test showed that after treatment with *Gymnadeniae* tubers, the serum (9.30 ± 4.18 nmol/ml, *P* < 0.01; and 13.71 ± 3.89 nmol/ml) and gastric (1.10 ± 1.19 nmol/ml, *P* < 0.05; and 0.96 ± 0.69 nmol/ml) MDA contents could be decreased in rats. The gastric ulcer index, MDA content in the serum and gastric ulcer index of the model group were 0.44 ± 0.18 cm, 14.17 ± 4.88 and 3.81 ± 5.35 nmol/ml, respectively. The above results suggest that the tubers exert a degree of inhibition on gastric ulcers that were induced by the hydrochloride-ethanol mixture (Lin, 2009). Jiang et al. (2009) also studied the beneficial effect of the tuber of *G. conopsea* on acute gastric ulcers in rats that were induced by hydrochloride-ethanol solution (7.5 ml/kg for 3 days). After drug administration orally (2 and 1 g/kg) for 30 min, the gastric ulcer areas were decreased with inhibitions of 88.86 (*P* < 0.01) and 48.88 (*P* < 0.01); the inhibition by a commonly prescribed drug (ranitidine, 1.95 g/kg) was 91.12 (*P* < 0.01). The pathology of the gastric ulcers was markedly improved by these drugs (Table 4).

### Immunoregulatory activity

As a traditional folk medicine, the tuber of *G. conopsea* has been widely indicated to have good tonifying and immunoregulatory activities (Chinese Materia Editorial Committee, State Chinese Medicine Administration Bureau, 2002; Li et al., 2006). In 2014, Shang et al. (2014) first evaluated the immunoregulatory functions of the polysaccharide of the tuber of *G. conopsea* in mice. After administering orally the polysaccharide (100, 50, and 10 μg/g) and distilled water for 28 days, the serum lysozyme content (104.8 ± 7.8, 102.5 ± 2.8, and 100.1 ± 7.3, all *P* < 0.01), thymus index (0.42 ± 0.03, *P* < 0.01; 0.35 ± 0.01, *P* < 0.01; and 0.25 ± 0.02) and spleen index (0.92 ± 0.07, *P* < 0.01; 0.85 ± 0.04, *P* < 0.01; and 0.79 ± 0.10, *P* < 0.05) were significantly improved relative to that of the control group (86.7 ± 2.8 mg/L, 0.22 ± 0.02, and 0.72 ± 0.05 mg/g). In the delayed type hypersensitivity (DTH) test, the drugs could promote the genesis of DTH and induce ear swelling, especially for the high dose group (30.0 ± 0.4 mg, *P* < 0.01) compared with the control group (15.6 ± 0.5 mg). The polysaccharides could significantly improve the phagocytic function of mouse macrophages, and the phagocytosis rates were 69.6 ± 1.3 (*P* < 0.01), 58.6 ± 0.5 (*P* < 0.05) and 49.2 ± 1.0 (*P* < 0.05) compared with that of the control group at 40.8 ± 0.7. These results suggest that the polysaccharide has a marked immunoregulatory function (Table 4).

### Anti-aging activity

In 2013, Si and Liu (2013) studied the anti-aging effects and mechanisms of the Shouzhangshen-37 pill on subacute aging in mice (the tuber of *G. conopsea* is the primary component of this pill). The subacute aging model mice were induced by injecting D-galactose (120 mg/kg) once per day for 7 weeks. High (2.4 g/kg), middle (1.2 g/kg), and low (0.6 g/kg) doses of Shouzhangshen-37 pills and a common drug (VE, 38.9 mg/kg) were also administered i.g. for 7 weeks. After the treatment, the anti-aging effects and mechanisms were evaluated by observing learning and memory ability with step-through tests, the indexes of the brain, thymus, and spleen were calculated by measuring the SOD, CAT, and MDA contents of brain tissues and observing the pathomorphism changes in mouse cerebral tissue by HE coloration. The results showed that compared with the model group, the drugs could enhance the memory ability and decrease the number of mistakes within 300 s (1.54 ± 0.53, *P* < 0.05; 1.26 ± 0.42, *P* < 0.01; and 1.71 ± 0.82, *P* < 0.05) compared with that of the model group (2.11 ± 0.81) and VE group (1.26 ± 0.42, *P* < 0.01). In addition, the brain tissue index (12.97 ± 1.89 mg/g, *P* < 0.05; 13.39 ± 1.39 mg/g, *P* < 0.01; and 13.05 ± 2.35 mg/g, *P* < 0.05), spleen (4.53 ± 0.66 mg/g, *P* < 0.05; 4.76 ± 0.82 mg/g, *P* < 0.01; and 4.58 ± 0.65 mg/g, *P* < 0.05), and thymus (1.11 ± 0.12 mg/g, *P* < 0.05; 1.21 ± 0.20 mg/g, *P* < 0.01; and 1.14 ± 0.25 mg/g, *P* < 0.05;) were improved compared with the model group (10.43 ± 1.66, 3.63 ± 0.55, and 0.91 ± 0.18 mg/g). The results of the biochemical test showed that the SOD activity (170.8 ± 20.3 U/mg, *P* < 0.05; 180.6 ± 14.7 U/mg, *P* < 0.01; and 177.3 ± 20.1 U/mg, *P* < 0.05) and CAT activity (6.51 ± 0.22, 7.14 ± 0.72, and 6.32 ± 0.23 U/mg, all *P* < 0.05) were increased and MDA content (3.21 ± 0.98 nmol/mg, *P* < 0.05; 3.17 ± 0.73 nmol/mg, *P* < 0.01; and 3.36 ± 0.81 nmol/mg, *P* < 0.05) was decreased compared with that of the model group (156.9 ± 14.5, 5.24 ± 0.51 U/mg, and 4.31 ± 0.47 nmol/mg). These results suggest that the Shouzhangshen-37 pill has an anti-aging effect in subacute aging mice. The mechanism may enhance the antioxidant activity in brain tissue, decrease the MDA content in brain tissue, protect the brain nerve cells, and improve memory ability in mice (Table 4).

### Anti-hyperlipidemia activity

In 2013, Zhang et al. (2013) studied the effects of the tuber of *G. conopsea* ethanol extracts on the blood lipids and liver function of experimental hyperlipidemia rats. The results showed that after treating the hyperlipidemia model rats that had been induced by high-fat diets with ethanol extracts (at 5, 2.5, and 1.25 g/kg, i.g.), the TC and LDL-C serum contents of the model rats were not improved; however, the TG and HDL-C contents were decreased at 1.35, 0.89, 0.97 mmol/L (*P* < 0.01), and 0.49 (*P* < 0.05), 0.53 and 0.49 mmol/L (*P* < 0.05) compared with the model group (2.43 and 0.64 mmol/L), and the commonly prescribed drug lovastatin also showed markedly decreased TG and HDL-C content (*P* < 0.01). In addition, the ALT and AST activities in the serum were also inhibited at10.71, 10.82, 8.21 U/L (*P* < 0.05), 6.97 (*P* < 0.01), 13.60 (*P* < 0.05), and 18.02 U/L compared with that of the model group, and the commonly prescribed drug results were 8.53 (*P* < 0.05) and 10.38 (*P* < 0.01). These results suggest that the tuber could reduce blood lipids and protect the liver function in experimental hyperlipidemia rats (Table 4).

### Antianaphylaxis activity

In 2004, Matsuda et al. (2004) studied the effects of the methanolic extract of the *G. conopsea* tuber. The results showed an anti-allergic effect on passive cutaneous anaphylaxis reactions in the ears of mice. The inhibitory effects of the principal constituents on β-hexosaminidase release, which acts as a marker of degranulation in RBL-2H3 cells, were examined, and five phenanthrenes (gymconopin B, 4-methoxy-9,10-dihydrophenanthrene-2,7-diol, 1-(4-hydroxybenzyl)-4-methoxyphenanthrene-2,7-diol, 1-(4-hydroxybenzyl)-4-methoxy-9,10-dihydrophenanthrene-2,7-diol, blestriarene A) and six dihydrostilbenes (gymconopin D, batatasin III, 3′-*O*-methylbatatasin III, 3,3′-dihydroxy-2-(4-hydroxybenzyl)-5-methoxybibenzyl, 3′,5-dihydroxy-2-(4-hydroxybenzyl)-3-methoxybibenzyl, and 3,3′-dihydroxy-2,6-bis(4-hydroxybenzyl)-5-methoxybibenzyl) were found to inhibit antigen-induced degranulation by 65.5–99.4% at 100 μM in RBL-2H3 cells (Table 4).

### Anti-silicosis activity

Silicosis is an important occupational disease caused by the inhalation of silica dust, and it is characterized by lung interstitial fibrosis. In 2007 and 2008, Wang et al. (2007, 2008) studied the effects of silica exposure on collagen synthesis in rat lungs and mechanisms of anti-oxidative stress by using a 60% ethanol extract of the tuber of *G. conopsea*. Silicotic animal models were established by surgically directing the tracheal instillation of silica into rat lungs. After administering the ethanol extract orally (8 g/kg, per day), the rats were sacrificed and the samples were collected to assay the relative index at 7, 14, 21, 28, and 60 days. The results showed that the extract could reduce the lung/body weight ratio of rats (8.6, 6.99, *P* < 0.01; 7.25, *P* < 0.05; 7.97, *P* < 0.05; and 9.75 mg/g, *P* < 0.05) relative to the model group (11.04, 9.28, 9.11, 11.98, and 13.91 mg/g), and the extract could also improve pathological changes in the lung. This treatment could also ameliorate silica-induced pulmonary fibrosis by decreasing the type I and type III collagen-positive area percentage in the lungs of rats exposed to silica at different time points. These studies also indicated that the ethanol extract could decrease the MDA content (4.78, *P* < 0.01; 5.39, *P* < 0.05; 5.48, *P* < 0.05; 5.29, *P* < 0.05; and 4.35 mmol/L) and increase the SOD (302.67, 243.95, *P* < 0.01; 293.38, *P* < 0.05; 277.74, *P* < 0.05; and 243.36 KU/L) and GPx (2199.58, 2359.34, *P* < 0.01; 2538.66, *P* < 0.01; 2422.41, *P* < 0.05; and 2298.04 U/L) activities relative to that of the model group (6.43, 6.88, 6.98, 6.12, and 5.82 mmol/L, 314.84, 183.62, 219.41, 218.44, and 226.50 KU/L, and 2489.50, 1015.34, 1227.83, 1814.90, and 1867.38 U/L). In 2007, Zeng et al. (2007) found that the ethanol extract could reduce the TNF-α integral optical density (86.86, 105.1, 122.09, 108, and 94.88, all *P* < 0.01) of rat lungs compared with that of the model group (116.98, 140.1, 220.19, 140.9, and 110.9) at 7, 14, 21, 28, and 60 days, respectively. Before and after treating with a 60% ethanol extract, Chen (2008) studied the differential gene expression profiles of rat lung tissue in the early stage exposure to silica. After treating with the extract (8 g/kg, per day, i.g.), there were 308 and 231 up-regulated and down-regulated genes among a total of 539 available genes compared with that of the model group. Up-regulated pathways might be associated with cell adhesion molecules, notch signaling pathways, and leukocyte transendothelial migration and cell communication. Down-regulated pathways might be related to genes in the linkage of complementary and coagulation cascades, hematopoietic cell lineages, urea cycle and Alzheimer's disease. The chip results were verified by using real-time PCR for SOD and HMOX genes with the same trends. Thus, the investigators indicated that the alcohol extract may inhibit silicosis progress during early exposure periods and attenuate airs acculitis and fibrosis of the lung. The results also showed that the ethanol extract (8 g/kg, per day, i.g.) has the reverse effect on silicosis, and this process involved the cathepsin D precursor SEC14-like protein 3 and peroxiredoxin-1 (Chen, 2009; Table 4).

### Other activities

In 2008, Zi (2008) studied the anti-cancer activity of certain compounds from the tuber of *G. conopsea*. The results showed that the IC_50_-value of (−)-4-[β-D-glucopyranosyl-(1→4)-β-D-glucopyranosyloxy] benzyl alcohol against human hepatoma cancer cells (Bel7402) was 3.818 μM compared with the commonly prescribed drug camptothecine at 12.5 μM. At a concentration of 10^−6^ mol/L, gastrodin was active against serum deprivation-induced SH-SY5Y apoptosis.

At a dose of 5 mg/kg (i.p.), dactylorhin B, coelovirin E and (2R,3S)-1-(4-hydroxybenzyl)-2-hydroxy-4-(4-hydroxybenzyl)-2-isobutyltartrate could improve mouse memory deficits induced by scopolamine with a neuroprotective effect. Coelovirin E and (2R,3S)-1-(4-hydroxybenzyl)-2-hydroxy-4-(4-hydroxybenzyl)-2-isobutyltartrate could extend the latent time for the step-down test in mice with improvements of 65 and 106%, respectively (Zi, 2008; Table 4).

## Preparations and qualitative and quantitative analysis

*G. conopsea* has been widely used as a folk medicine in the treatment or prevention of diseases for thousands of years in China, but it has not been listed in the Chinese pharmacopeia because there is a lack of scientific quality standards to control the quality of the tuber since 1977 (Committee for the Pharmacopoeia of P.R. China, 1977, 2000; Chinese Materia Editorial Committee, State Chinese Medicine Administration Bureau, 2002). Recently, experts have attempted to formulate the proper quality standard by analyzing the active compounds and determining the contents of plants from different habitats with different chromatographic equipment.

Now, as a strategy to control the quality of folk medicines, chemical fingerprint analyses have been accepted by the WHO (1991), State Food and Drug Administration of China (2000), and other authorities, which has been recognized as a rapid and reliable method of identifying and qualifying herbal medicines. Cai et al. (2006) first developed a high-performance liquid chromatography-diode array detection-tandem mass spectrometry (HPLC-DAD-MS^*n*^) method for the chemical fingerprint analysis and rapid identification of major compounds in *G. conopsea* tubers. An HPLC separation was performed by using a linear gradient at room temperature (20°C) and a flow rate of 0.7 ml/min with an Inertsil C_18_ ODS-3 column. The gradient elution started with a methanol: water mixture (20:80, v/v), and the methanol content was increased to 100% within 60 min; the detection wavelength for fingerprint analysis was 270 nm. Adenosine, 4-hydroxybenzyl alcohol, 4-hydroxybenzyl aldehyde, dactylorhin B, loroglossin, dactylorhin A, and militarine were well-separated and identified from the tuber of 10 samples, which were collected from Sichuan, Qinghai, and Hebei Provinces and Tibet Autonomous Region of China and Nepal. Xue et al. (2013) also studied the fingerprint of *G. conopsea* by using HPLC. Their samples were separated on a Kromasil C_18_ (4.6 × 250 mm, 5 μm) column and gradient-eluted with a mixture of methanol and water (0.04% phosphoric acid) at a flow rate 0.7 ml/min; the detection wavelength was set at 222 nm, and the column temperature was 30°C. The results showed that 13 common peaks were selected, and the method validation met the technical requirements of a fingerprint. The similarity of 12 samples from the different regions of the Xizang Autonomous Region and Qinghai Province was 0.904–0.989. The above two methods are simple, practical and reliable. The combined use of the two fingerprints confirmed the identification and quality assessment of *G. conopsea*. In 2009, after investigating the optimal extraction method, Li et al. (2009) firstly developed a HPLC method for the simultaneous quantification of five glucosyloxybenzyl 2-isobutylmalates in the tubers of *G. conopsea*, which was collected at five regions in China. The optimal extraction conditions was the direct reflux with 70% ethanol for 1 h, and the compounds are separated on an Agilent Hydrosphere C18 (150 × 4.6 mm i.d., 5 μm) column using a mobile phase of acetonitrile-water including 0.3% acetic acid (adjusted with 36% acetic acid) with gradient elution at a flow rate of 1.0 mL/min. Detection is set at a UV wavelength of 221.5 nm. The recovery of the method is 97.7–101.0%, and linearity (*r* > 0.9998) is obtained for all the analytes. The results showed that the contents of dactylorhin B, dactylorhin E, loroglossin, dactylorhin A, and militarine were 1638.28, 100.19, 279.91, 717.22, and 101.78 mg/g (Lijiang City, Yunan Province), 665.82, 92.12, 248.15, 507.65, and 138.57 mg/g (Weixian City, Hebei Province), 707.84, 85.64, 322.63, 705.70, and 137.30 mg/g (Kangding City, Sichuan Province), 2207.30, 204.46, 604.81, 2213.39, and 228.04 mg/g (Xizang Autonomous Region), and 4073.89, 156.81, 3060.63, 2230.67, and 563.56 mg/g (Xining City, Qinghai Province). Yang et al. (2009) and Yang (2010) determined the contents of four marker compounds from the tubers of *Gymnadenia conopsea* (L.) R. Br. by HPLC, and the tubers were collected from 24 regions, including the Sichuan Province, Xizang Autonomous Region, Jilin Province, Heilongjiang Province, Qinhai Province, and other regions in China. At a detection wavelength of 222 nm, the mobile phases of methanol (A) and water (B) were used in a gradient elution at 0.7 ml/min. The initial condition was 15–20% A in 0–10 min, which increased to 45% A in 20 min and 70% A in 35 min. The results showed that the highest contents of dactylorhin A, dactylorhin B, loroglossin, and militarine from the rhizomes of24 different regions were 1.428 and 1.638 mg/g from Lulang County in the Xizang region and 1.148 and 0.521 mg/g from Jilin Province. In 2011, the gastrodin contents were assayed by HPLC, and the results showed that the highest content was 9.113 mg/g in Tongde County of Qinghai Province, China, followed by 7.785 mg/g (Beinagou region, Qinghai Province), 4.881 mg/g (Huangnan Zeku region, Qinghai Province), 4.309 mg/g (Guoluo region, Qinghai Province), 4.285 mg/g (Batang Grassland, Qinghai Province), 3.895 mg/g (Nangqian County, Qinghai Province), 3.719 mg/g (Maixiu Forest Farm, Qinghai Province), 3.242 mg/g (Jinyingtan, Qinghai Province), 3.024 mg/g (Linzhi County, Xizang Autonomous Region), 3.103 mg/g (Chengduo, Qinghai Province), 2.734 mg/g (Bomi County, Xizang Autonomous Region), and 2.268 mg/g (Beishan Forest Farm, Huzhu County, Qinghai Province; Xue et al., 2011).

Meanwhile, the content of four marker constituents of the tubers with the climatic factors were determined and analyzed by HPLC in 2014. The results showed that the samples collected from Milin County in the Xizang Autonomous Region had the highest gastrodin content at 2.725 mg/g, and the Shennongjia region of Hubei Province had lowest content at 0.374 mg/g. The samples collected from Kangding County of Sichuan Province, Aba region of Sichuan Province and Naqu County of the Xizang Autonomous Region had the highest contents of adenosine, 4-hydroxybenzyl alcohol and 4-hydroxybenzaldehyde at 1.485, 1.505, and 1.048 mg/g, respectively. These findings suggest that the average temperature and annual average variations in monthly temperature change along with seasonal variations, and the highest temperature, variations in the scope of the annual temperature, average temperature of the most humid season, average temperature of the hottest season, annual rainfall, rainfall during the driest season, rainfall during the coldest season, and latitude and altitude of the habitat can have an effect on the active ingredient content and quality of the tuber of *G. conopsea* (Zhang et al., 2014).

The study results also indicated that the samples collected from Huzhu County of Qinghai Province had the highest contents of gastrodin (4.9242 mg/g), dactylorhin A (8.2274 mg/g), and militarine (7.4645 mg/g; Yao and Lin, 2014).

## Toxicity

In 2007, Bai and Zheng (2007) evaluated the toxicity of the tuber of *G. conopsea*. In acute toxicity test, after administering the tuber (1.00, 2.15, 4.64, and 10 g/kg, i.g.) to mice and rats, the behavior, and growth of the animals did not change after 2 weeks compared to the control group (*P* > 0.05), and the LD_50_ was more than 10 g/kg. The genotoxicity of the medicine was then studied, and the results showed that the rates of bone marrow and sperm abnormalities were not changed compared with that of the control group, but the sperm abnormality rates of the group administered a common drug (cyclophosphamide, 30 mg/kg) changed markedly (*P* < 0.01). And after administering the tuber (1.67, 3.33, and 6.67 g/kg, i.g) to rats for 30 days, the parameters of blood routine, biochemical indexes, organ coefficient, and organ pathology of rats haven't marked changed compared the control group (*P* > 0.05). So they thought that the tuber didn't present the apparent toxicity.

## Conclusions and future perspectives

As an important traditional medicine in China, the tuber of *G. conopsea* has been historically used in a number of clinical applications. However, lack of scientific quality standard to control the quality of the tuber has been hampered to the development of this plant. Up to now although investigators have begun studying and developing the quality of the tuber and its preparations by analyzing the marker compounds and determining the contents of plants from different habitats, a scientific method to control the quality of these products has not yet been developed and approved by government. Thus, a characteristic chemical and biological index should be established to monitor and evaluate quality of samples and maintain their clinical and pharmaceutical stability, and the method of accurately controlling the quality of the tuber in traditional medicine and preparations should be studied further.

With the excavation and abundant use of *G. conopsea*, as well as the over-grazing and disorder tourism resulted in the habitat destruction, the resources of this plant were rapid decreased. At the same time, because of the low reproductive capacity of this plant, once the resources and habitat have been destroyed, the species does not recover easily. In 2000, *G. conopsea* has been listed in the grade Π section of endangered species (Gesang and Gesang, 2010). Recently, investigators have begun studying and developing methods of cultivating this plant, such as tissue culture and artificial breeding, but this technology is still not applied in industry (Bao et al., 2008). Therefore, the sustainable use of *G. conopsea* is necessary to investigate. Of course, along with the decrease of resources, the studies on the aerial part should be paid more attention, especially for finding new active compounds and bioactivities.

In a word, phytochemical and pharmacological studies of *G. conopsea* have received great interest, and an increasing number of extracts and active compounds have been isolated that have demonstrated tonifying activity, anti-viral activity and immunoregulatory activity, among others. However, validating the correlations of the ethnomedicinal uses and pharmacological effects should be carried out further, and the toxicity of this plant also should be studied systematically. Meanwhile, the poor quality control, decreased resources, the increasing gap between more experience-based traditional uses and less evidence-based clinical trials for the tuber of *G. conopsea* has created challenges. Therefore, this plant should be studied and developed further, especially in the resource conservation.

## Author contributions

XS and JZ conceived the review; XS, XG, XM wrote the manuscript; HP collected the literatures; and YL edited the manuscript. All the authors read and approved the final version of the manuscript.

### Conflict of interest statement

The authors declare that the research was conducted in the absence of any commercial or financial relationships that could be construed as a potential conflict of interest.

## Footnotes

^1^The Plant List, version 1 (Accessed January 01, 2010).

^2^The Flora of China.

## Abbreviations

BSA: Bovine Serum Albumin
CAT: Catalase
DNA: Deoxyribonucleic Acid
DPPH: 1,1-diphenyl-2-picrylhydrazyl
DTH: Delayed type hypersensitivity
HBsAg: Hepatitis B Surface Antigens
HBV: Hepatitis B virus
HDL-C: High Density Lipoprotein-Cholesterol
HE: Hemoglobin Electrophoresis
HIV-1: Human Immunodeficiency Virus-1
HPLC: High-performance Liquid Chromatography
HPLC-DAD-MS^*n*^: High-performance Liquid Chromatography-diode Array Detection-tandem Mass Spectrometry
HPSEC-MALLS/RID: High Performance Size Exclusion Chromatography Coupled with Multi-angle Laser Light Scattering/refractive Index Detector
IC_50_: 50% inhibition concentration
LD_50_: Median Lethal Dose
LDL-C: Low Density Lipoprotein-Cholesterol
M_w_: Weight-average Molecular Weight
MDA: Malondialdehyde
MeOH: Methyl Alcohol
PACE: Polysaccharide Analysis by Carbohydrate Gel Electrophoresis
PCR: Polymerase Chain Reaction
SOD: superoxide dismutase
TC: Total Cholesterol
TCM: Traditional Chinese Medicine
TG: Triglyceride
WHO: World Health Organization.
